# Pre‐treatment comorbidities, C‐reactive protein and eosinophil count, and immune‐related adverse events as predictors of survival with checkpoint inhibition for multiple tumour entities

**DOI:** 10.1002/cam4.5919

**Published:** 2023-04-21

**Authors:** Tarun Mehra, Kanchan Dongre, Maria Boesing, Patricia Frei, Claudia Suenderhauf, Alfred Zippelius, Joerg D. Leuppi, Andreas Wicki, Anne B. Leuppi‐Taegtmeyer

**Affiliations:** ^1^ Department of Oncology Medical University Clinic Kantonsspital Baselland Liestal Switzerland; ^2^ Department of Oncology & Hematology University Hospital Zürich, University of Zurich Zürich Switzerland; ^3^ Department of Clinical Pharmacology & Toxicology University Hospital Basel, University of Basel Basel Switzerland; ^4^ Department of Patient Safety University Hospital Basel Basel Switzerland; ^5^ Medical University Clinic Kantonsspital Baselland Liestal Switzerland; ^6^ Department of Oncology University Hospital Basel, University of Basel Basel Switzerland

**Keywords:** checkpoint inhibitor, comorbidity, CRP, immunotherapy, melanoma, non‐small cell lung cancer

## Abstract

**Background:**

The development of immune‐related adverse events (irAEs) may be associated with clinical efficacy of checkpoint inhibitors (CPIs) in patients with cancer. We therefore investigated the effect of irAEs and pre‐treatment parameters on outcome in a large, real‐life patient cohort.

**Methods:**

We performed a single‐centre, retrospective, observational study including patients who received CPIs from 2011 to 2018 and followed until 2021. The primary outcome was overall survival, and the secondary outcome was the development of irAEs.

**Results:**

In total, 229 patients with different tumour entities (41% non‐small cell lung cancer [NSCLC], 29% melanoma) received a total of 282 CPI treatment courses (ipilimumab, nivolumab, pembrolizumab or atezolizumab). Thirty‐four percent of patients developed irAEs (of these 17% had CTCAE Grade ≥3). Factors independently associated with mortality were pre‐treatment CRP ≥10 mg/L (hazard ratio [HR] 2.064, *p* = 0.0003), comorbidity measured by Charlson comorbidity index (HR 1.149, *p* = 0.014) and irAEs (HR 0.644, *p* = 0.036) (age‐adjusted, *n* = 216). Baseline eosinophil count ≤0.2 × 10^9^/L was a further independent predictor of mortality (age‐, CRP‐, CCI‐ and irAE‐adjusted HR = 2.252, *p* = 0.002, *n* = 166). Anti‐CTLA‐4 use (*p* < 0.001), and pre‐treatment CRP <10 mg/L were independently associated with irAE occurrence (*p* = 0.037).

**Conclusions:**

We found an independent association between irAE occurrence and improved overall survival in a real‐life cohort spanning multiple tumour entities and treatment regimens. Pre‐treatment comorbidities, CRP and eosinophil count represent potential markers for predicting treatment response.

## Graphical abstract

In this real‐life study of survival with checkpoint inhibitor therapy for multiple tumour entities, we observed an independent association of pre‐treatment comorbidities, CRP and eosinophil count, and occurrence of irAE with improved overall survival. These parameters represent promising markers for predicting treatment response.

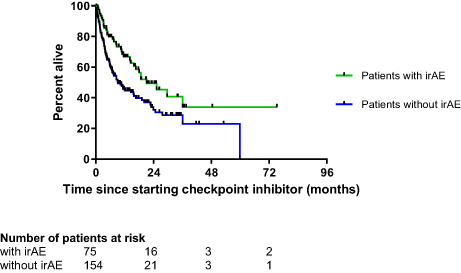

## 1 INTRODUCTION

Immune checkpoint blockade presents a remarkable advance in the treatment of numerous tumour entities.^1^, ^2^, ^3^, ^4^ The new treatment options have opened the possibility of continuous remission for conditions considered fatal a few years ago.

There are two main groups of immune checkpoint inhibitors, namely inhibitors of cytotoxic T‐lymphocyte‐associated protein 4 (CTLA‐4) and inhibitors of programmed cell death 1 ligand 1 (PD‐L1) or its receptor, programmed cell death protein 1 (PD‐1).^2^ Their specific mechanisms of action via blockade of negative regulators of T‐cell activation, proliferation and migration can result in the occurrence of immune‐related adverse events (irAEs).^5^

The incidence of irAEs due to treatment with the CTLA‐4‐antibody ipilimumab is approximately 60% for all events and 10%–30% for common toxicity criteria for adverse events (CTCAE) Grade 3–4 events.^5^ The incidence of total and severe irAEs during treatment with ipilimumab appears to be dose dependent.^6^ The incidence of irAEs is lower with PD‐1 and PD‐L1 inhibitors, with total irAEs of any CTCAE Grade occurring in approximately 20%, and Grade 3–4 irAEs in approximately 10% of patients.^6^ So far, clear evidence of dose‐dependence for PD‐1 and PD‐L1 inhibitor‐induced irAEs is lacking. Combination therapy greatly increases the risk of irAEs, with severe irAEs (irAE ≥ CTCAE Grade 3) occurring in more than 50% of patients.^6^ IrAEs also tend to occur sooner after treatment onset with combination therapy.^6^

There is evidence for a possible link between irAEs and checkpoint‐inhibitor treatment efficacy. Development of irAEs has been associated with improved survival in patients receiving immune checkpoint inhibitors as a treatment for non‐small‐cell lung cancer (NSCLC),^7^ renal cell carcinoma,^8^ melanoma^9^ and gastrointestinal cancer.^10^ There are, however, some studies which do not support a correlation between the occurrence of irAEs and improved survival.^11^, ^12^

The aim of this study was to investigate the effect of irAEs on survival in a large, real‐life patient cohort treated with CPIs for multiple tumour entities. We also investigated whether baseline (pre‐treatment) parameters were predictive of the development of irAEs and of survival.

## 2 MATERIALS AND METHODS

A single‐centre, retrospective, observational study was performed. All patients receiving checkpoint inhibitor therapy at this centre between January 2011 and January 2018, and who did not refuse their authorisation for their clinical data to be used for scientific purposes as defined in the general consent form, were included in our study. The primary outcome was overall survival and the secondary outcome was the development of irAEs. Local data‐archiving and retrieval processes meant that data regarding CPI treatment and irAEs was only available for the time period spanning January 2011 until April 2018. Patients were followed up until censoring on 30 April 2018 (first censor date) and until censoring on 31 December 2021 (second censor date, extended study period) or until death. The median follow‐up time for survivors was calculated.

Results were analysed with descriptive statistics using Microsoft® Excel 2016 (Microsoft Corporation). Correlation was assessed with Spearman's rho (*ρ*). Survival analysis was performed by plotting Kaplan–Meier curves with GraphPad Prism 8.0.2 for Windows (GraphPad Software). Significance in differences of survival (hazard ratio [HR]) were analysed with the log‐rank test. We refrained from imputation and used complete datasets for calculation. Values of laboratory measurements below the validated detection threshold were assumed to be 0. Multivariate logistic regression was used to identify and model the effect of risk factors for irAE occurrence, and multivariate Cox regression was used to analyse predictors of survival (both with R version 4.1.2^13^). Interacting terms were additionally investigated in the multivariate analyses. In order to determine whether independent predictors remained associated with survival over time, we re‐examined them using the extended study period censor date December 2021. With an alpha level fixed at 0.05, we considered results to be significant if *p* was <0.05. Additional information about data collection can be found in the supplementary file.

The study was approved by the regional ethics committee (2017‐02329) and was performed in accordance with the Declaration of Helsinki as well as in adherence to STROBE recommendations. Cases of irAE were reported to the national drug regulatory authority (Swissmedic) in accordance with the national pharmacovigilance laws.

## 3 RESULTS

### 3.1 Patient characteristics

Table 1 summarises patient characteristics. Only a few patients were treatment‐naïve before receiving CPI (eight patients). Eighty per cent of patients suffered from documented co‐morbid conditions, 17 of whom (7%) suffered from autoimmune conditions. Among the 225 patients for whom the medication records could be retrieved, the median number of concurrent medications for the treatment of comorbid conditions was 3 (IQR 4). None of these comedications were anti‐tumoural agents. Of the 183 patients for which smoking history could be retrieved, only a minority (28%) had never smoked. Most of the smokers were heavy smokers, 86% having a smoking history of at least 20 pack‐years.

**TABLE 1:** Patient characteristics (*N* = 229).

Parameter (number for whom data available)	Number (%), median [range] or mean ± SD
Total number of patients	229
Female	75 (33%)
Age (years) at start of checkpoint inhibitor treatment [range]	65 [18–91]
Age ≥ 50 years	201 (88%)
Body mass index (kg/m^2^, *n* = 221)	24.5 [15.0–43.2]
Patients with body mass index ≥30 kg/m^2^	36 (16%)
Patients with autoimmune diseases	17 (7%)
Charlson comorbidity index; median	9 [1–15]
Charlson comorbidity index <5	8 (3%)
Charlson comorbidity index 5–10	180 (79%)
Charlson comorbidity index 11–15	41 (18%)
Charlson comorbidity index >15	0
Patients without co‐medication (*n* = 225)	27 (12%)
Median number of co‐medications (*n* = 225)	3 [0–11]
Patients with history of smoking (current/ex) (*n* = 183)	131 (72%)
Median pack years (*n* = 112)	45 [1–100]
Pre‐treatment CRP (mg/L, *n* = 216)	31.9 ± 50.1
Pre‐treatment eosinophil count (*n* = 166)	0.208 ± 0.354
Indication for checkpoint inhibitor treatment	
Non‐small cell lung cancer	95 (41%)
Melanoma	66 (29%)
Renal cell carcinoma	15 (6%)
Head and neck squamous cell carcinoma	13 (6%)
Hepatocellular carcinoma	9 (4%)
Urothelial carcinoma	8 (4%)
Hodgkin‐lymphoma	2 (1%)
High microsatellite instability colorectal cancer	2 (1%)
Other indications	19 (8%)
PDL‐1 expression (*n* = 75)	
Positive	42 (56%)
Negative	33 (44%)
Median % PDL‐1 positivity in positive tumours (*n* = 38) [IQR]	30 [55]
Median time between diagnosis and first CPI treatment (months)	12.5 [0.6–227]

Abbreviations: CRP, C‐reactive protein; IQR, interquartile range; PDL‐1, programmed death ligand‐1.

### 3.2 Checkpoint inhibitor therapy characteristics

A total of 281 CPI treatment lines were recorded, with an average of 1.2 CPI treatment lines per patient. Details of the CPI treatment lines are given in Table S1, which also provides the median number of cycles per CPI given in the first treatment line. Figure S1 shows the first‐line CPI treatments and the tumour entities for which they were applied.

### 3.3 Immune‐related adverse events

Seventy‐five patients (33%) developed irAEs (CTCAE Grades 1–5) and 154 (67%) did not. A total of 137 irAEs were recorded. IrAEs per CPI compound and toxicity type along with median time to development of irAEs are summarised in Table S2.

IrAEs occurred the most frequently under combination therapy with ipilimumab/nivolumab (1.2 irAEs per treatment line) and the least frequently with nivolumab alone (0.31 irAEs per treatment line) (Table 2). Seven events occurred with atezolizumab (1 patient), 22 with ipilimumab (14 patients), 44 with nivolumab (34 patients), 34 with pembrolizumab (23 patients) and 30 events with ipilimumab + nivolumab (14 patients). A total of 23 events were classified as being at least severe (CTCAE Grade ≥3), of which one was a CPI‐related death due to colitis under ipilimumab. Two cases of colitis under ipilimumab + nivolumab combination therapy were classified as life‐threatening. IrAEs CTCAE ≥3 were more frequent with anti‐CTLA‐4‐use compared to anti‐PD‐L1 or anti‐PD‐1 use. Twenty‐seven percent and 23% of irAEs were CTCAE ≥3 for ipilimumab monotherapy and ipilimumab/nivolumab combination therapy, respectively. For atezolizumab, nivolumab and pembrolizumab monotherapy, these figures were 0%, 9% and 18%, respectively.

**TABLE 2:** Frequency of immune‐related adverse events (irAEs) according to checkpoint‐inhibitor (CPI) substance.

Checkpoint inhibitor	Number of CPI treatment courses	Number of irAEs	Number of irAEs per CPI course
Nivolumab	144	44	0.31
Pembrolizumab	66	34	0.52
Ipilimumab	30	22	0.73
Nivolumab + ipilimumab	24	30	1.2
Atezolizumab	16	7	0.44
Atezolizumab + ipilimumab	1	0	0
Total	281	137	0.49

Overall 54 (72%) patients developed early irAEs and 21 (28%) patients developed late irAEs. The time‐to‐event of irAEs per CPI class, severity and anatomical location are depicted in Figure S2. Cutaneous adverse events occurred the soonest at a median of 40 days, with joint toxicity occurring the latest, at a median of 112 days (Table S2). Serious irAEs (CTCAE ≥3) tended to occur sooner (median 48 days) in comparison to moderate or mild irAEs (median of 61 days of time to occurrence, *p* = ns). IrAEs under Anti‐CTLA‐4‐immunotherapy (ipilimumab) occurred earlier than irAEs under PD‐L1/PD‐1 immunotherapy (median time to event 43.5 vs. 78 days, *p* = 0.007). IrAEs under ipilimumab monotherapy also showed dose‐dependency. The mean ipilimumab dose received by patients with IrAEs was 4 ± 2.86 mg/kg body weight (range 3–10) compared to 2.9 ± 0.29 mg/kg (range 2–3) received by those without irAEs.

The systemic treatments of the 137 irAEs are summarised in Table S3. Sixty‐three per cent were treated with systemic steroids, of which 10 episodes of gastrointestinal irAE were additionally treated with biological agents.

We assessed independent associations of pre‐treatment variables recorded at the start of CPI treatment with the development of irAEs. Table 3 shows the results of a binary multivariate logistic regression analysis with the occurrence of any irAE as the dependent variable and age, comorbidity (assessed by total number of points on the Charlson comorbidity index), anti‐CTLA‐4 use, baseline eosinophil count >0.2 × 10^9^/L and baseline CRP <10 mg/L as independent variables. Anti‐CTLA‐4‐use (OR 4.96, *p* < 0.001) and baseline CRP <10 mg/L (OR 2.16, *p* = 0.037) were independent predictors of the development of irAEs. While autoimmune diseases were present in a higher proportion of patients who went on to develop irAEs compared to those who did not develop irAEs (9% vs. 3%), this difference was not statistically significant. Lastly, we also analysed the development of irAE according to tumour type (Table S4).

**TABLE 3:** Binary multivariate logistic regression predicting immune‐related adverse events (*N* = 166).

	Odds ratio	95% CI		*p* value
		Lower	Upper	
Age (years)	1.019	0.984	1.055	0.295
Total CCI‐Points	0.927	0.757	1.134	0.461
Anti‐CTLA4 use	4.955	2.116	11.639	<0.001
Baseline EOS >0.2 × 10^9^/L	1.013	0.466	2.204	0.973
Baseline CRP < 10 mg/L	2.158	1.048	4.440	0.037
Constant	0.136	0.021	0.879	0.036

Abbreviations: CCI, Charlson comorbidity index; CTLA4, cytotoxic T lymphocyte‐associated protein 4; EOS, eosinophils, CRP, C‐reactive protein.

### 3.4 Follow‐up and survival

The median follow‐up time for survivors was 13.5 months (IQR 17.0 months) at the April 2018 censoring date and 54 months (IQR 22 months) at the December 2021 censoring date. There were 169 deaths during the entire study period and the median survival time was 14.5 months (Figure S3, which also shows the survival according to tumour type).

### 3.5 Baseline data and overall survival

We determined whether baseline factors which were independently associated with irAE until April 2018 (shown in Table 3) were also independently associated with survival until December 2021. Anti‐CTLA‐4 use was not examined in this analysis due to its strong correlation with irAE and treated tumour entity.

**FIGURE 1:**
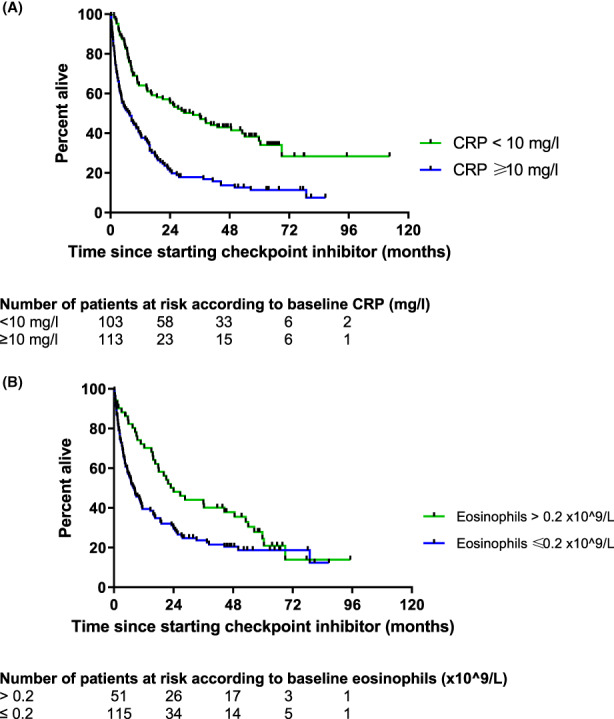
(A) Kaplan–Meier survival curves of patients with (blue line) and without (green line) high baseline C‐reactive protein (*N* = 216). High baseline C‐reactive protein was defined as ≥10 mg/L. Log‐rank *p* < 0.0001, hazard ratio 2.31 (95% CI 1.68–3.16). (See Figures S4 and S5 for sub‐analyses according to tumour type.) Patients were followed until 31st December 2021. (B) Kaplan–Meier survival curves of patients with (green line) and without (blue line) high baseline eosinophil count (EOS) under checkpoint‐inhibitor treatment (*N* = 166). High baseline eosinophil count was defined as >0.2 × 10^9^/L. Log‐rank *p* = 0.014, hazard ratio 0.63 (95% CI 0.44–0.89). Patients were followed until 31st December 2021.

Patients with an elevated CRP at baseline ≥10 mg/L had a substantial and significant poorer survival than patients with a low CRP at baseline (*p* < 0.0001, hazard ratio 2.31, 95% CI 1.68–3.16) (Figure 1). This result was also seen in the main tumour subset NSCLC (*p* = 0.041, hazard ratio 1.69, 95% CI 1.05–2.7) (Figure S4) and in the melanoma subset (Figure S5), although statistical significance was not reached (*p* = 0.1, hazard ratio 1.92, 95% CI 0.73–5.08). In addition, patients with a higher eosinophil count at baseline (>0.2 × 10^9^/L) had improved survival in comparison to patients with a low eosinophil count at baseline (*p* = 0.014, hazard ratio 0.63 [95% CI 0.44–0.80]) (Figure 1). Increasing comorbidity defined by the Charlson comorbidity index was inversely correlated with survival (*ρ* = −0.145, 95% confidence interval 0.02–0.27, *p* = 0.014).

In a multivariate Cox survival analysis corrected for age at start of checkpoint inhibitor treatment, total Charlson comorbidity Index points, eosinophil count ≤0.2 × 10^9^/L and CRP ≥10 mg/L were independently associated with poorer survival (Table 4).

**TABLE 4:** Age‐adjusted multivariate Cox regression models for death during the study period.

Study period until	No. patients	Parameter	Hazard ratio	95% CI		*p*‐value
				Lower	Upper	
December 2021	166	Age at start of CPI treatment (years)	0.99	0.97	1.01	0.395
		Total CCI‐points	1.13	1.02	1.25	0.017
		Baseline EOS ≤0.2 × 10^9^/L	1.53	1.08	2.33	0.031
		Baseline CRP ≥ 10 mg/L	2.10	1.38	2.91	6.75 × 10^−5^
April 2018	166	Age at start of CPI treatment (years)	0.998	0.976	1.021	0.865
		Total CCI‐points	1.151	1.016	1.303	0.027
		Baseline EOS ≤0.2 × 10^9^/L	2.252	1.362	3.717	0.002
		Baseline CRP ≥ 10 mg/L	2.058	1.313	3.226	0.002
		irAE	0.774	0.488	1.227	0.276
April 2018	216	Age at start of CPI treatment (years)	0.993	0.974	1.013	0.513
		Total CCI‐Points	1.149	1.029	1.283	0.014
		Baseline CRP ≥ 10 mg/L	2.064	1.399	3.045	0.0003
		irAE	0.644	0.427	0.971	0.036

Abbreviations: CPI, checkpoint inhibitor, CCI, Charlson comorbidity index, in points, EOS, eosinophils, CRP, C‐reactive protein, irAE, immune‐related adverse events.

### 3.6 irAE and overall survival

**FIGURE 2:**
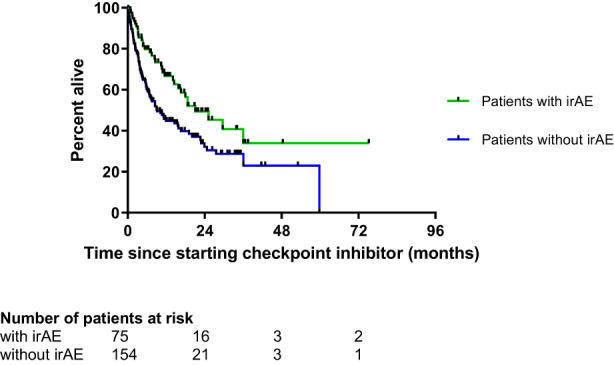
Kaplan–Meier survival curves of patients with (green line) and without (blue line) immune‐related adverse events (irAE) during checkpoint‐inhibitor treatment (*N* = 229). Log‐rank *p* = 0.006, hazard ratio 0.58 (95% CI 0.40–0.83) (median follow‐up of survivors 13.5 months, IQR 17.0 months). Patients were followed until 30th April 2018.

We analysed the association between the occurrence of irAEs and survival until the first censorship date (30 April 2018). There were 125 deaths, 91 (59%) in the group without an irAE (*n* = 154) and 34 (45%) events in the group with irAEs (*n* = 75). Patients who experienced at least one irAE had a significant improved survival over those patients without irAEs (hazard ratio 0.58 [95% CI 0.40–0.83], *p* = 0.006) (Figure 2). Survival according to irAE and tumour type is shown in Figure S6. When the 14 patients who received a PD‐1‐inhibitor in combination with a CTLA‐4‐inhibitor as their first‐line treatment were excluded, the log‐rank test yielded a HR of 0.60 [95% CI 0.41–0.88] (*p* = 0.015).

Patients with mild or moderate first irAEs (CTCAE Grades 1 and 2) had similar survival compared to patients with severe first irAEs (*p* = 0.7, Figure S7). Patients who experienced skin, endocrine and joint toxicity as their first irAE had better survival compared to patients without irAEs (*p* = 0.0067, Figure S8). There was no difference in survival according to receipt of systemic treatments for irAEs (Figure S9).

We went on to assess the association of baseline parameters (age at start of CPI treatment, CRP, eosinophil count and CCI) and irAE with survival until April 2018 by performing a multivariate Cox regression. While CRP <10 mg/L, eosinophil count >0.2 × 10^9^/L and a low total CCI at baseline were independently associated with improved survival, age and the occurrence of irAE were not (Table 4). However, because patients without baseline eosinophil measurements (*N* = 63) were excluded, the model lost statistical power. We therefore developed a model with age, baseline CRP, CCI and irAE as covariates (*N* = 216, Table 4). In this model, higher CCI points and higher CRP were independently associated with poorer survival, whereas the occurrence of irAE was independently associated with better survival.

## 4 DISCUSSION

In this study of irAE and survival after CPI therapy in a large, real‐life patient cohort, we found that anti‐CTLA‐4 use and a pre‐treatment CRP <10 mg/L were predictors of the occurrence of irAE. Furthermore, pre‐treatment CRP <10 mg/L, eosinophils >0.2 × 10^9^/L, having fewer comorbidities and the development of irAE were independently associated with improved survival. Baseline parameters were predictive of survival during the extended study period (median follow‐up 14.5 months), indicating that these might be useful prognostic indicators of how well a patient will respond to CPI therapy.

Predictive markers for the development of irAEs are not that well established. Possible mechanisms implicated in the development of irAEs include cytotoxic T‐cell activation, increased autoantibody production due to B cell activation, molecular mimicry and off‐target toxicity, pro‐inflammatory cytokine production and environmental factors such as the gut microbiome.^14^ Recently, elevated TNF‐alpha, or IFN‐alpha‐2 at baseline as well as LCP1 and ADP‐dependent glucokinase have been identified as biomarkers associated with the development of irAEs.^15^, ^16^ These biomarkers, however, are not routine investigations and therefore likely to be of limited use in daily clinical practice. Kartolo and colleagues identified a history of autoimmune disease, use of CTLA‐4‐inhibitors and poor kidney function as risk factors for the development of irAEs.^17^ Our analysis was able to confirm the role of anti‐CTLA‐4 CPI as a predictor for the development of irAEs and supports the finding of poor kidney function (a parameter included in CCI) and autoimmune disease as additional risk factors. Unlike the study by Kartolo and colleagues,^17^ we also investigated the association of baseline CRP and eosinophils with the development of irAE and found that lower CRP, but not eosinophils was associated with subsequent irAE. CRP—an acute phase reactant as well as a marker of chronic inflammation—has been found to be associated with a poorer prognosis in several studies of patients with solid tumours and is proposed to be an indicator of a high tumour load.^18^ Being components of the immune microenvironment, eosinophils modulate tumour initiation and progression and play an anti‐tumorigenic role in several neoplasia including melanoma and colorectal cancer.^19^ There is already indirect evidence that a lower baseline CRP is associated with the development of irAE. Lauwyck et al. report that a rise in baseline CRP predicted severe irAEs.^20^ The association between a rising CRP from baseline and the occurrence of irAEs is plausible, because inflammatory processes due to immune deregulation cause irAEs.^5^ However, to our knowledge, the finding of low pre‐treatment CRP predicting the occurrence of irAE is novel. Further, large‐scale studies are needed to confirm or refute our observation.

On the contrary, the association between higher CRP at baseline and poorer treatment outcome has already been established. Higher CRP levels were associated with poorer survival in a retrospective analysis from samples obtained prospectively from the Checkmate‐067 trial.^21^ These results confirm those from other studies.^22^, ^23^, ^24^ Our Kaplan–Meier survival analyses corroborate the findings. Moreover, we were able to show that a pre‐treatment elevated CRP ≥10 mg/L was a significant predictor for death, even when controlling for age and comorbidity level. The underlying pathomechanism may be that a higher tumour burden increases systemic inflammation, and that a higher tumour burden per se as well as an elevated inflammatory state poorly affects response to anti‐tumoural therapy and therefore survival.^25^ This hypothesis is supported by the findings of Simeone et al. demonstrated the association between increasing CRP under CPI treatment and poor overall survival (median 4.6 vs. 16.9 months in patients with stable or decreased CRP).^26^ Interestingly, in the present study, not only melanoma but also NSCLC patients responded less well to CPI treatment if pre‐treatment CRP levels are elevated.

The role of an elevated eosinophil count as a prognostic marker for survival in cancer patients under immunotherapy has previously been suggested,^27^ which subsequently has been corroborated in larger prospective trials.^28^, ^29^ Our results are consistent with these findings. However, pre‐treatment eosinophil count was not associated with the occurrence of irAE, which suggests a role as a prognostic marker that is independent of irAE. In a study of 17 patients who developed immune‐related adrenal insufficiency during CPI treatment, Takayasu et al. found that an increase in eosinophil count from baseline (but not baseline eosinophil count) was associated with irAEs.^30^ This was not, however, observed in a further 34 patients with thyroid irAEs in the same study.^30^ In a further study, Chu et al. found an association between baseline absolute eosinophil count >0.125 × 10^9^ cells/L and the development of interstitial pneumonitis during CPI treatment in 300 patients (54 of whom developed interstitial pneumonitis).^31^ The fact that we did not observe an association between baseline eosinophil counts and irAE may therefore be due to the fact that different irAEs were examined together. Chu and colleagues also report a better clinical outcome in patients with eosinophil counts >0.125 × 10^9^ cells/L, consistent with our findings.^31^

The association between the development of irAEs and improved survival has been shown for multiple cancer types and CPI treatments.^8^, ^9^, ^20^, ^32^, ^33^, ^34^, ^35^, ^36^ We were able to corroborate this association in our study. However, our hazard ratio for overall survival of 0.58 was higher than ratios reported in the literature. Grangeon et al. reported a HR for survival of 0.29 in a NSCLC cohort,^33^ Ricciuti et al. a HR of 0.33 also in a NSCLC cohort^34^ and Indini et al. of 0.39 in a melanoma cohort.^35^ Because all studies are retrospective, differences in study design do not seem to explain the discrepancies, nor do the demographic profiles, as in all studies most patients were over 60 years old. Because NSCLC and melanoma are the two most common tumour entities by far, the difference can also not be solely explained by a variation in tumour type. PD‐L1 expression might have differed between cohorts. The aforementioned studies included only anti‐PD‐1/anti‐PD‐L1 CPIs and no anti‐CTLA‐4‐CPIs. In our study, irAE was independently associated with overall survival when the effect of age, comorbidities and baseline CRP were controlled for, implying that the development of irAE confers good anti‐tumoural response to CPI therapy. However, when a smaller subset of patients for whom baseline peripheral blood eosinophil counts were available was examined (*n* = 166), irAE was no longer independently associated with overall survival, implying that the development of irAE is a weaker predictor than baseline eosinophil count, CRP and CCI. Like other investigators, we also found that the use of systemic steroids in the management of irAEs did not reduce the treatment effect of CPIs.^12^, ^20^, ^37^

Our study also has some limitations further to its inherent sample‐size‐, missing data‐ and unobserved confounder‐constraints. First, our analysis is retrospective. Second, we were not always able to retrieve PD‐L1 expression from the medical records. Third, time series for laboratory values were not consistently available. Lastly, stratified analyses were only possible in a limited fashion. Nevertheless, we report data from a well‐characterised single‐centre cohort, and our results are consistent with those from other, similar‐sized studies. This strengthens the validity of our findings.

In conclusion, we corroborate the association between irAE occurrence and improved overall survival in a real‐life cohort spanning multiple tumour entities and treatment regimens. Pre‐treatment parameters, namely comorbidities, serum CRP concentration and peripheral blood eosinophil count represent routinely available potential markers for predicting treatment response.

## AUTHOR CONTRIBUTIONS

**Tarun Mehra:** Conceptualization (equal); formal analysis (equal); methodology (equal); writing —original draft (lead). **Kanchan Dongre:** Data curation (supporting); formal analysis(supporting); investigation (equal); project administration (equal); writing—review and editing (supporting). **Maria Boesing:** Formal analysis (equal); writing—review and editing (supporting). **Patricia Anina Frei:** Data curation (lead); formal analysis (supporting); investigation (equal); project administration (equal); writing—review and editing (supporting). **Claudia Suenderhauf:** Conceptualization (supporting); project administration (supporting); writing—review and editing (supporting). **Alfred Zippelius:** Methodology (supporting); resources (equal); supervision (equal); writing—review and editing (supporting). **Joerg Leuppi:** Resources (supporting); writing—review and editing (supporting). **Andreas Wicki:** Conceptualization (equal); formal analysis (supporting); methodology (supporting); resources (equal); supervision (equal); writing—review and editing (supporting). **Anne Leuppi‐Taegtmeyer:** Conceptualization (lead); data curation (supporting); formal analysis (equal); investigation (supporting); methodology (lead); project administration (lead); supervision (equal); writing—original draft (supporting); writing—review and editing (lead).

## FUNDING INFORMATION

The authors declare there were no funding sources.

## CONFLICT OF INTEREST STATEMENT

The authors declare there were no conflicts of interest.

## ETHICS STATEMENT

The study was approved by the regional ethics committee (2017‐02329).

## Supporting information


**Data S1:** Supporting Information

## Data Availability

The data that support the findings of this study are available from the corresponding author upon reasonable request.
