# Supplementary material for: Pre‐treatment comorbidities, C‐reactive protein and eosinophil count, and immune‐related adverse events as predictors of survival with checkpoint inhibition for multiple tumour entities
Source: Cancer Med. 2023 Apr 21;12(11):12253–62. doi: 10.1002/cam4.5919 (PMC10278511; doi:10.1002/cam4.5919)
Supplement: Supplementary file 1 — Data S1: Supporting Information [file CAM4-12-12253-s001.docx]

**Supplement**

**Pre-treatment comorbidities, C-reactive protein and eosinophil count, and immune-related adverse events as predictors of survival with checkpoint inhibition for multiple tumour entities**

**Running title:** **Checkpoint inhibition, CRP, irAE and survival**

Tarun Mehra MD^1,2^, Kanchan Dongre MD^3,4^, Maria Boesing MD^5^, Patricia Frei MSc^3^, Claudia Suenderhauf MD PhD^3^, Alfred Zippelius MD^6^, Joerg D Leuppi MD PhD^5^, Andreas Wicki MD PhD^1,2*^, Anne B Leuppi-Taegtmeyer MD PhD^3,4,5*^

^1^ Department of Oncology, Medical University Clinic, Kantonsspital Baselland, Liestal, Switzerland

^2^ Department of Oncology & Hematology, University of Zurich and University Hospital Zürich, Zürich, Switzerland

^3^ Department of Clinical Pharmacology & Toxicology, University and University Hospital Basel, Basel, Switzerland

^4^ Department of Patient Safety, University Hospital Basel, Basel, Switzerland

^5^ Medical University Clinic, Kantonsspital Baselland, Liestal, Switzerland

^6^ Department of Oncology, University and University Hospital Basel, Basel, Switzerland

*Shared last authorship

**Supplementary methods**

The study population was identified by searching the hospital pharmacy database for all patients who had received at least one full cycle of checkpoint inhibitor therapy. Data were collected by manually reviewing paper and electronic medical records. Laboratory Data were retrieved from hospital data silos. Data points were transcribed to Microsoft Excel spreadsheets for further analyses.

Variables collected included the indication of CPI treatment, date of diagnosis of the underlying malignancy, previous cancer treatments, date and cause of death (if deceased), and demographics (age at start of CPI treatment, gender, weight and height). Co-administered drugs, allergies and comorbidities were recorded. The degree of polymorbidity was calculated using the Charlson comorbidity index (CCI) that predicts 10-year survival in patients with comorbidities. The index scores range from 0 to 37; with higher scores predicting a shorter survival (1). Parameters used in the score are age category (per decade from the age of 50 years), diabetes, liver disease, solid tumour, acquired immunodeficiency syndrome, moderate to severe chronic kidney disease, congestive heart failure, myocardial infarction, chronic obstructive pulmonary disease, peripheral vascular disease, cerebrovascular disease, dementia, hemiplegia, connective tissue disease, leukaemia, malignant lymphoma and peptic ulcer disease. Treatment information including compound name, start and stop date, administered dose and number of doses administered were collated. These data were also collated for subsequent CPI courses, if they were given. irAE type and severity, classified according to CTCAE criteria (2), were recorded, in addition to date of onset and resolution, treatment and outcome. irAEs occurring during the first 12 weeks of CPI treatment were classified as “early irAEs”; irAEs occurring after 12 weeks were classified as “late irAEs”. CTCAE grades 3 and above were classified as “severe”. Pre-treatment (“baseline”) laboratory values for haematological (haemoglobin, leucocytes, platelets, eosinophils and neutrophils) and biochemical parameters (sodium, potassium, creatinine, estimated creatinine clearance and C-reactive protein), defined as a laboratory sample taken within 12 weeks prior to the first dose of the checkpoint inhibitor, were analysed. When counting the number of cycles for combination CPI treatment, we counted the number of cycles of the compound which was used for longer, in case of discrepancies.

**Table S1** Treatment characteristics of the patients (n = 229)

| **Treatment characteristic** | **Number of patients (%)** | **Median cycle number; IQR** | |
| --- | --- | --- | --- |
| Number of CPI treatment lines |  |  |  |
| 1 line | 183 (80) | n/a |  |
| 2 lines | 40 (17) | n/a |  |
| 3 lines | 6 (3) | n/a |  |
|  |  |  |  |
| CPI given for first treatment line |  |  |  |
| Nivolumab | 121 (53) | 5.5; 18* |  |
| Pembrolizumab | 53 (23) | 4; 6 |  |
| Ipilimumab | 26 (11) | 2.5; 2 |  |
| Nivolumab + Ipilimumab | 15 (7) | 2; 2 |  |
| Atezolizumab | 14 (6) | 4; 4.75 |  |
| Atezolizumab + Ipilimumab | 0 (0) | n/a |  |

n/a not applicable, * data available for 118 patients

**Table S2** Immune-related adverse events per checkpoint-inhibitor compound and toxicity type.

| irAE |  | All compounds | Nivolumab | Pembrolizumab | Atezolizumab | Ipilimumab | Ipilimumab + Nivolumab |
| --- | --- | --- | --- | --- | --- | --- | --- |
| All irAEs | Total number (number CTCAE ≥ 3) | 137 (23) | 44 (4) | 34 (6) | 7 (0) | 22 (6) | 30 (7) |
|  | Median time to event (days; IQR) | 58 (29-173) | 79.5 (36-214) | 64 (32.5-166.5) | 147 (63.5-536) | 43.5 (28.5-62.25) | 45 (15-235) |
|  |  |  |  |  |  |  |  |
| Cutaneous | Total number (number CTCAE ≥ 3) | 43 (8) | 11 (1) | 10 (3) | 3 (0) | 6 (1) | 13 (3) |
|  | Median time to event (days; IQR) | 40 (17.5-160) | 68 (27-359) | 49 (33.75-61.75) | 147 (range 42-532) | 28 (15-38.75) | 27 (10-85) |
|  |  |  |  |  |  |  |  |
| Endocrine | Total number (number CTCAE ≥ 3) | 26 (1) | 9 (0) | 7 (0) | 1 (0) | 5 (1) | 4 (0) |
|  | Median time to event (days; IQR) | 80 (50-210) | 57 (39-81) | 147 (77-204) | 868 | 63 (58-98) | 189 (42-335.25) |
|  |  |  |  |  |  |  |  |
| Gastrointestinal | Total number (number CTCAE ≥ 3) | 26 (9, 1 death) | 7 (2) | 5 (0) | 1 (0) | 7 (4, 1 death) | 6 (3) |
|  | Median time to event (days; IQR) | 56 (28.5-116.25) | 93 (38-187.5) | 124 (65-403) | 21 | 45 (30-55.5) | 55 (48-64) |
|  |  |  |  |  |  |  |  |
| Joint toxicity | Total number (number CTCAE ≥ 3) | 15 (2) | 6 (0) | 6 (2) | 2 (0) | 0 | 1 (0) |
|  | Median time to event (days; IQR) | 112 (31-248.5) | 118 (52.75-134.5) | 105 (39 -303) | 313 (range 85-540) |  | 1 |
|  |  |  |  |  |  |  |  |
| Pulmotoxicity | Total number (number CTCAE ≥ 3) | 12 (1) | 6 (1) | 3 (0) | 0 | 2 (0) | 1 (0) |
|  | Median time to event (days; IQR) | 60 (19.5-156.5) | 141 (49-190) | 15 (range 14-78) |  | range 21-182 | 42 |
|  |  |  |  |  |  |  |  |
| Hepatotoxicity | Total number (number CTCAE ≥ 3) | 11 (2) | 3 (0) | 2 (1) | 0 | 1 (0) | 5 (1) |
|  | Median time to event (days; IQR) | 53 (33-115) | 76 (range 28-154) | 38 (range 34-42) |  | 63 | 53 (32-285) |
|  |  |  |  |  |  |  |  |
| Other* | Total number (number CTCAE ≥ 3) | 4 (0) | 2 (0) | 1 (0) |  | 1 (0) |  |
|  | Median time to event (days; IQR) | 74 (range 33-313) | 177 (range 108-245) | 108 |  | 33 |  |
|  |  |  |  |  |  |  |  |

Time to event: time between start of check-point inhibitor and occurrence of adverse event, in days. CPI: check point inhibitor. CTCAE: common toxicity criteria of adverse events. IQR: interquartile range. irAE: immune-related adverse events. *Two cases of myocarditis (pembrolizumab and ipilimumab), one case each of encephalitis and vasculitis (nivolumab)

**Table S3** Systemic treatment of the 137 irAEs

| **Medication** | **Number (% of total)** | **Mean dose ± SD (mg)** |
| --- | --- | --- |
| None | 51 (37) |  |
| Oral steroids | 66 (48) |  |
| Prednisone | 55 (40) | 71.5 ± 44.4 |
| Methylprednisolone | 3 (2) | 67 ± 29 |
| Dexamethasone | 1 | unknown |
| Hydrocortisone | 6 (4) * | 35 ± 10 (2 missing) |
| Intravenous steroids | 20 (15) |  |
| Methylprednisolone | 18 (13) | 134 ± 36 (1 missing) |
| Hydrocortisone | 2 (1) ** | 100 (1 missing) |
| Additional biological agents*** | 10 (7) |  |
| Infliximab | 7 (5) |  |
| Vedolizumab | 2 (2) |  |
| Adalimumab | 1 (1) |  |
| Additional mycophenolate | 3 (2) |  |
|  |  |  |

*endocrine irAEs in 5 cases **endocrine irAEs in both cases ***given for gastrointestinal irAEs

**Table S4** Development of irAE according to tumor type

| irAE |  | All patients | NSCLC | Melanoma | Other |
| --- | --- | --- | --- | --- | --- |
| All irAEs | Number of patients (CTCAE ≥ 3) | 75 (14) | 23 (4) | 38 (7) | 14 (3) |
|  | Median time to event (days; IQR) | 42 (87) | 66 (139) | 32 (73) | 34 (36) |
|  |  |  |  |  |  |
| Cutaneous | Number (CTCAE ≥ 3) | 29 (5) | 6 (1) | 16 (3) | 7 (1) |
|  | Median time to event (days; IQR) | 31 (52) | 140 (331) | 28 (29) | 25 (31) |
|  |  |  |  |  |  |
| Endocrine | Number (CTCAE ≥ 3) | 12 (1) | 5 (0) | 6 (1) | 1 (0) |
|  | Median time to event (days; IQR) | 61 (72) | 57 (26) | 90 (136) | 39 |
|  |  |  |  |  |  |
| Gastrointestinal | Number (CTCAE ≥ 3) | 13 (5) | 4 (1) | 7 (3) | 2 (1) |
|  | Median time to event (days; IQR) | 52 (97) | 194 (280) | 45 (102) | 52 (0.5) |
|  |  |  |  |  |  |
| Joint toxicity | Number (CTCAE ≥ 3) | 7 (1) | 2 (0) | 3 (0) | 2 (1) |
|  | Median time to event (days; IQR) | 33 (83) | 86 (53) | 29 (216) | 42 (28) |
|  |  |  |  |  |  |
| Pulmotoxicity | Number (CTCAE ≥ 3) | 8 (1) | 4 (1) | 3 (0) | 1 (0) |
|  | Median time to event (days; IQR) | 60 (78) | 62 (63) | 78 (109) | 6 |
|  |  |  |  |  |  |
| Hepatotoxicity | Number (CTCAE ≥ 3) | 3 (1) | 1 (1) | 2 (0) | none |
|  | Median time to event (days; IQR) | 29 (7) | 42 | 29 (0.5) |  |
|  |  |  |  |  |  |
| Other* | Number (CTCAE ≥ 3) | 3 (0) | 1 (0) | 1 (0) | 1 (0) |
|  | Median time to event (days; IQR) | 40 (140) | 313 | 33 | 40 |

**Supplementary Figures**

**Figure S1** First-line checkpoint-inhibitor substance per tumor entity (N=229 patients)

%: % of total (n=229). CPI: check point inhibitor. CRC: colorectal cancer. Hodgkin: Hodgkin lymphoma. MSI: microsatellite instability. NSCLC: non-small-cell lung cancer. SCLC: small-cell lung cancer.

**
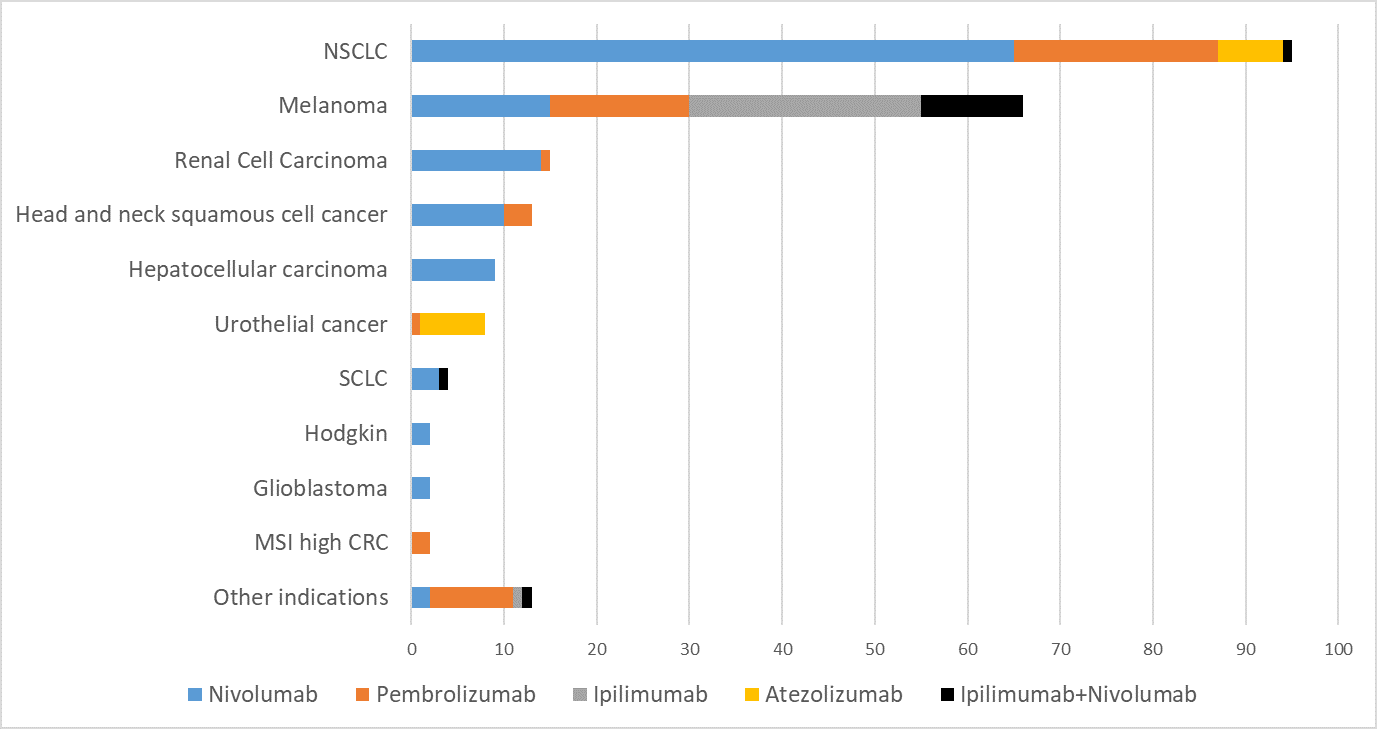
**

**Figure S2:** Empirical distribution function of immune-related adverse events (N = 137) by A) checkpoint inhibitor compound class, B) irAE severity and C) anatomical location

**A**

PD-L1/PD-1: Pembrolizumab, Nivolumab, Atezolizumab (N=85 events). CTLA4: Ipilimumab (N=22 events). Combination: Ipilimumab/Nivolumab (N=30 events).

**B**

Mild: CTCAE Grade 1; Moderate: CTCAE grade 2; Severe: CTCAE grade 3-4.

**C**

**Figure S3** Kaplan-Meier survival analysis until December 31^st^ 2021 (N = 229) **A** all patients and **B** patients grouped according to tumor type. NSCLC = non small-cell lung cancer

**A**

| **Number of patients at risk** | | | |  |  |  |
| --- | --- | --- | --- | --- | --- | --- |
|  | 229 | 83 | 48 | | 11 | 2 |

**B**

**Figure S4** Kaplan Meier survival curves of non-small-cell lung cancer patients with and without high baseline C-reactive protein (N=89). High baseline C-reactive protein defined as ≥10 mg/l. Log-rank P=0.041, hazard ratio 1.69, 95% CI 1.05-2.7. Patients were followed until 31^st^ December 2021.

**Figure S5** Kaplan Meier survival curves of melanoma patients with and without high baseline C-reactive protein (N=64). High baseline C-reactive protein (CRP) defined as ≥10 mg/l. Log-rank P = 0.1, hazard ratio 1.92, 95% CI 0.73 - 5.08. Patients were followed until 31^st^ December 2021.

**Figure S6** Kaplan Meier survival curves according to irAE and tumor type. Patients were followed until 30^th^ April 2018. **A** Patients with melanoma (n = 66), log-rank P = 0.57, hazard ratio 0.8, 95% CI 0.37 – 1.7. **B** Patients with NSCLC (n = 95), log-rank P = 0.2, hazard ratio 0.66, 95% CI 0.37 – 1.18 **C** Patients with other tumor types, log-rank P = 0.46, hazard ratio 0.76, 95% CI 0.37 – 1.53.

**A**

**B**

**C**

**Figure S7** Kaplan Meier survival curves of patients with no, mild/moderate or severe irAEs (N=227). (log-rank P=0.7 mild/moderate vs. severe). Patients were followed until 30^th^ April 2018.

| **Number of patients at risk** | | | |  |  |
| --- | --- | --- | --- | --- | --- |
| Severe irAE | 14 | 5 | 2 | | 2 |
|  |  |  |  | |  |
| Mild/mod irAE | 59 | 12 | 2 | | 1 |
|  |  |  |  | |  |
| No IrAE | 154 | 20 | 3 | | 1 |

**Figure S8** Kaplan Meier survival curves according to first irAE type (N=229). (log-rank “No irAE” vs. “Cutaneous, Endocrine, Joint” P=0.0067, “No irAE” vs. “Colitis, Hepatitis, Pneumonitis, Other” P = 0.21, “Cutaneous, Endocrine, Joint” vs. “Colitis, Hepatitis, Pneumonitis, Other” P = 0.4). Patients were followed until 30^th^ April 2018.

| **Number of patients at risk** | | | |  | |  | |  |
| --- | --- | --- | --- | --- | --- | --- | --- | --- |
| Cutaneous, endocrine, joint | 48 | 10 | | 3 | | 2 | |  |
|  |  |  | |  | |  | |  |
| Colitis, hepatitis, pneumonitis | 27 | 6 | | 1 | | 1 | |  |
|  |  |  | |  | |  | |  |
| No IrAE | 154 | 20 | | 3 | | 1 | |  |

**Figure S9** Kaplan Meier survival curves according to treatment of first irAE (N=229). (log-rank “Systemic treatment” vs “No systemic treatment“ P = 0.4). Patients were followed until 30^th^ April 2018.

| **Number of patients at risk** | | | |  | |  | |  |
| --- | --- | --- | --- | --- | --- | --- | --- | --- |
| Systemic treatment | 47 | 12 | | 2 | | 2 | |  |
|  |  |  | |  | |  | |  |
| No systemic treatment | 28 | 5 | | 2 | | 1 | |  |

1. Charlson ME, Pompei P, Ales KL, MacKenzie CR. A new method of classifying prognostic comorbidity in longitudinal studies: development and validation. J Chronic Dis. 1987;40(5):373-83.

2. US Department of Health and Human Services. Common Terminology Criteria for Adverse Events v.5.0. 2017; <https://ctep.cancer.gov/protocoldevelopment/electronic_applications/docs/ctcae_v5_quick_reference_5x7.pdf>. Last accessed May 2022.
